# Probabilistic strain optimization under constraint uncertainty

**DOI:** 10.1186/1752-0509-7-29

**Published:** 2013-03-29

**Authors:** Mona Yousofshahi, Michael Orshansky, Kyongbum Lee, Soha Hassoun

**Affiliations:** 1 Department of Computer Science, Tufts University, Medford, MA, USA; 2 Department of Electrical and Computer Engineering, University of Texas at Austin, Austin, TX, USA; 3 Department of Chemical and Biological Engineering, Tufts University, Medford, MA, USA

**Keywords:** Enzyme activity modification, Flux capacity, Uncertainty, Chance-constrained optimization

## Abstract

**Background:**

An important step in strain optimization is to identify reactions whose activities should be modified to achieve the desired cellular objective. Preferably, these reactions are identified systematically, as the number of possible combinations of reaction modifications could be very large. Over the last several years, a number of computational methods have been described for identifying combinations of reaction modifications. However, none of these methods explicitly address uncertainties in implementing the reaction activity modifications. In this work, we model the uncertainties as probability distributions in the flux carrying capacities of reactions. Based on this model, we develop an optimization method that identifies reactions for flux capacity modifications to predict outcomes with high statistical likelihood.

**Results:**

We compare three optimization methods that select an intervention set comprising up- or down-regulation of reaction flux capacity: CCOpt (Chance constrained optimization), DetOpt (Deterministic optimization), and MCOpt (Monte Carlo-based optimization). We evaluate the methods using a Monte Carlo simulation-based method, MCEval (Monte Carlo Evaluations). We present two case studies analyzing a CHO cell and an adipocyte model. The flux capacity distributions required for our methods were estimated from maximal reaction velocities or elementary mode analysis. The intervention set selected by CCOpt consistently outperforms the intervention set selected by DetOpt in terms of tolerance to flux capacity variations. MCEval shows that the optimal flux predicted based on the CCOpt intervention set is more likely to be obtained, in a probabilistic sense, than the flux predicted by DetOpt. The intervention sets identified by CCOpt and MCOpt were similar; however, the exhaustive sampling required by MCOpt incurred significantly greater computational cost.

**Conclusions:**

Maximizing tolerance to variable engineering outcomes (in modifying enzyme activities) can identify intervention sets that statistically improve the desired cellular objective.

## Background

In recent years, increasingly sophisticated computational methods have been developed to identify optimal genetic modifications to achieve a desired metabolic engineering objective. The problem of identifying optimal genetic modifications can be expressed in terms of operating state variables such as reaction flux, and control (decision) variables such as the presence or absence of gene expression. The optimal design “tunes” these variables such that the solution meets the engineering objective while satisfying several constraints reflecting physicochemical considerations, experimental observations and assumptions about the physiology of the cell or organism. Due to biological variability [1,2], stochastic effects associated with gene expression, and imprecision in engineering implementation, it is questionable that enzyme levels can be precisely tuned to exactly match the target values calculated using computational design tools. More likely, the target enzyme levels, and thus the corresponding reaction flux capacities, can only be achieved with a finite degree of uncertainty. Addressing uncertainty at the design stage is a challenging issue that has become increasingly important not only for engineering biological systems, but also man-made systems such as electronic devices. Indeed, the past decade has witnessed a paradigm shift in design of electronics and computational design tools, where all modern electronic circuits are now designed to maximize tolerance to manufacturing and operational variations or to include tuning circuitry for post-manufacturing re-calibration. As metabolic engineering efforts progress from proof-of-principle to scaled-up manufacturing, computational methods to effectively address biological and engineering uncertainties at the design stage will become increasingly important in ensuring the identification of the most robustly optimal gene modifications.

The uncertainty in achieving targeted enzyme values suggests that the enzyme levels, and hence the corresponding flux carrying capacities (bounds), could be considered statistical distributions rather than fixed value parameters. In this statistical interpretation, a flux constraint in a conventional deterministic optimization problem represents the most conservative point in the flux capacity distribution, since a deterministic problem enforces all constraints with zero uncertainty. Although the deterministic approach affords relatively straightforward problem formulation and is most commonly practiced [3-5], this approach might lead to choosing an intervention set that may be far from optimal in a statistical sense. Alternatively, a sampling-based optimization approach (e.g. Monte Carlo sampling [6]), with the obvious caveat of being computationally intensive, probabilistically explores a possible space of enzyme activities, i.e. flux capacity distributions, and solves for an optimal intervention set for each sampled instance of flux capacities. Repeated sampling produces multiple intervention sets and a corresponding distribution of objective function values. Another alternative for incorporating uncertainties in an optimization problem is chance-constrained programming (CCP), which selects an optimal solution with a user-defined degree of probabilistic confidence in meeting constraints. Chance-constrained programming was first introduced in [7] to solve the problem of temporal planning when uncertainty is present. Since then, CCP has been utilized in numerous applications, including circuit sizing [8], soil conservation [9], ground water management [10], energy management [11], and molecular property optimization [12].

Current strain optimization methods generally seek to identify combinations of gene-level modifications that will result in an improvement of the desired cellular objective. These modifications are commonly gene deletions, but may be also up- or down-regulations of gene expression. A notable example of a computational method to identify gene knockouts is OptKnock [4]. This method uses bi-level programming to identify gene deletions that satisfy the coupled objectives of metabolite overproduction and biomass formation. Another gene deletion strategy is Genetic Design through Local Search (GLDS) [5], which employs a heuristic and flux balance analysis (FBA) to iteratively find sets of zero flux reactions (corresponding to gene deletions) that would result in the maximization of the target reaction flux. Other, related methods for large-scale problems involve metaheuristic approaches to iteratively improve a candidate set of gene deletions by generating and selecting variants of the candidate set via assessment of the objective function. An example of this approach is OptGene, which uses an evolutionary algorithm to improve the set of gene deletions with respect to an objective function [13].

Optimization methods have also been described to identify targets for gene expression modification. OptReg [3] is a constraint-based method that uses bi-level programming to determine which sets of genes should be amplified or down-regulated to satisfy a coupled pair of engineering and cellular objectives. Another class of computational strain design methods utilizes elementary mode (EM) analysis. One recent example is Computational Approach for Strain Optimization aiming at high Productivity (CASOP), which ranks reactions based on their contributions to the yield of desired product [14]. Another example is Flux Design, which selects reactions for up-regulation or deletion based on their correlation with the objective flux computed from EMs that contribute to the target product [15,16]. Despite increasing sophistication, these and other current computational strain design methods implicitly assume that reaction flux changes can be implemented precisely, and thus do not consider uncertainties as part of the problem formulation.

In this paper, we investigate three computational methods to address uncertainty in strain optimization. Specifically, we compare two probabilistic methods, CCP based optimization (CCOpt) and sampling based optimization (MCOpt), against deterministic optimization (DetOpt). The performance of each method is tested on two metabolic models for which enzyme level changes and corresponding flux capacity distributions are estimated either from kinetic parameters or steady-state flux data. The performance of the solutions, i.e. predicted target fluxes and corresponding intervention sets, is evaluated using Monte Carlo simulations (MCEval) designed to simulate the variable outcomes resulting from experimental implementation of the modifications specified by the optimization solutions.

## Methods

### Chance-constrained optimization (CCOpt)

**Figure 1:**
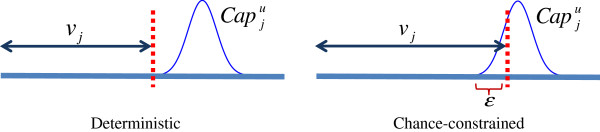
**Deterministic and chance-constrained interpretation of an upper bound on reaction flux.** The dotted lines represent the upper bound for the flux of a reaction *j* in a deterministic (left panel) and chance-constrained interpretation (right panel). The arrows show the flux ranges. If the upper bound is a random variable, the deterministic interpretation forces the flux *v*_*j*_ below the lowest value in the upper bound distribution. The chance-constrained interpretation allows *v*_*j*_ to exceed the lowest value in the upper bound distribution by some probability specified by the parameter *ε*.

Figure 1 illustrates the difference between a deterministic and probabilistic interpretation of an uncertain upper-bound constraint on the flux of reaction *j*. In the deterministic interpretation, the value of flux *v*_*j*_ of any feasible solution is enforced to be strictly less than all of the values in the upper-bound (flux capacity) distribution $\mathrm{Ca}p_{j}^{u}$. This yields the constraint:

(1) $$\text{Prob}\left\{v_{j}<\mathrm{Ca}p_{j}^{u}\right\}=1$$

In the probabilistic interpretation, the constraint is not always satisfied, i.e. there is a nonzero probability that flux *v*_*j*_ will be equal to or larger than some of the values in the distribution $\mathrm{Ca}p_{j}^{u}$. In the case of CCP, the constraint is relaxed by introducing a parameter *ε*, which reflects the confidence level for the probability that the solution satisfies the constraint:

(2) $$\text{Prob}\left\{v_{j}<\mathrm{Ca}p_{j}^{u}\right\}\geq 1-\varepsilon$$

To generalize the previous inequality to also consider the effects of up- or down-regulating the activity of an enzyme (e.g. through an adjustment in the expression of the gene that encodes the enzyme), we introduce two sets of binary decision variables $y_{j}^{u}$ and $y_{j}^{d}$. In this paper, we use the phrasing “up- or down-regulation” to describe engineering modifications that result in expression level changes of enzymes or groups of enzymes regardless of the method. A value of 1 indicates that the corresponding enzyme is up- or down-regulated, whereas a value of 0 indicates the corresponding enzyme expression is unchanged.

(3) $$\begin{array}{c} \text{Prob}\left\{v_{j}\leq \left(1-y_{j}^{u}\right)\left(1-y_{j}^{d}\right)\mathrm{SS}U_{j}\right)\\ \;\left(+y_{j}^{u}\left(1-y_{j}^{d}\right)\mathrm{Ca}p_{j}^{u}+y_{j}^{d}\left(1-y_{j}^{u}\right)\mathrm{Ca}p_{j}^{d}\right\}\geq 1-\varepsilon \end{array}$$

where 3, *SSU*_*j*_ denotes the reference (unmodified) state upper bound for reaction *j*. The fact that there are two random variables ($\mathrm{Ca}p_{j}^{u}$ and $\mathrm{Ca}p_{j}^{d}$) does not pose a challenge in solving such an inequality, as at most one of them will have a nonzero coefficient at a time. Mathematically, the sum of the two decision variables must be less than or equal to one ($y_{j}^{u}+y_{j}^{d}\leq 1$), which simplifies the above inequality into the following:

(4) $$\text{Prob}\left\{v_{j}\leq \mathrm{SS}U_{j}+y_{j}^{u}\left(\mathrm{Ca}p_{j}^{u}-\mathrm{SS}U_{j}\right)+y_{j}^{d}\left(\mathrm{Ca}p_{j}^{d}-\mathrm{SS}U_{j}\right)\right\}\geq 1-\varepsilon$$

**Figure 2:**
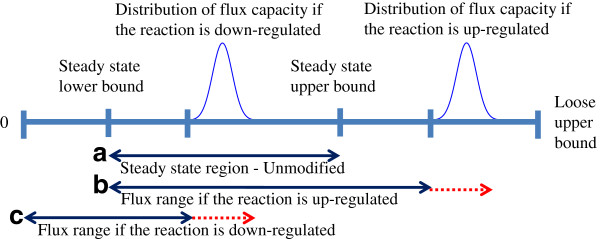
**Chance-constrained reaction flux bounds with or without enzyme level changes.** When there is no modification in the enzyme level, the flux for reaction *j* lies within the reference state range (**a**). When the reaction is up-regulated, the upper bound distribution shifts above the reference state upper bound (**b**). When the reaction is down-regulated, the upper bound distribution shifts below the reference state upper bound and the new (modified state) lower bound may also shift below the reference state lower bound (**c**). The red dashed lines in (b) and (c) show the range of possible flux capacity values equaling the spread of the capacity distributions.

A graphical illustration of the probabilistic constraints is shown in Figure 2. Down-regulating a reaction decreases the upper bound, or the flux capacity. It could also decrease the lower bound to zero. The capacity change could leave the flux unchanged or decrease it below the level of the reference (unmodified) state lower bound. Up-regulating a reaction increases the flux capacity, but does not affect the lower bound. The flux value could remain the same or rise above the reference state upper bound. In this study, we model the capacity change resulting from a gene expression modification as a probabilistic (rather than deterministic) event, which leads to a flux capacity distribution (dashed red lines).

Various approaches have been developed to solve CCP problems based on properties such as the distribution of random variables, linearity, and type (individual or joint) of the chance constraints [11]. One method to solve a CCP problem is to convert the probabilistic constraints (here, equation (4)) into their deterministic equivalents at their specified confidence level *ε*. This approach requires that the random variables of the problem are independent, and appear only in an exclusive linear form, such that the coefficients of all but one are always zero [17]. Our formulation meets all of these conditions; therefore, the chance constraints can be converted into their deterministic equivalents. Using the inverse of the cumulative distribution functions (CDF) for $\mathrm{Ca}p_{j}^{u}$ and $\mathrm{Ca}p_{j}^{d}$, inequality (4) can be reformulated as:

(5) $$\begin{array}{c} v_{j}\leq \mathrm{SS}U_{j}+y_{j}^{u}\left(F_{j,u}^{-1}\left(\varepsilon \right)-\mathrm{SS}U_{j}\right)\\ +y_{j}^{d}\left(F_{j,d}^{-1}\left(\varepsilon \right)-\mathrm{SS}U_{j}\right) \end{array}$$

where $F_{j,u}^{-1}$ and $F_{j,d}^{-1}$ denote the inverse CDFs of $\mathrm{Ca}p_{j}^{u}$ and $\mathrm{Ca}p_{j}^{d}$ respectively, which can be numerically calculated if needed.

Recasting the chance constraints into the equivalent deterministic constraints, the uncertain optimization problem of maximizing the flux of a desired product through gene up/down-regulation operations can be formulated for a system of arbitrary size consisting of *N* metabolites and *M* reactions. Without loss of generality, reversible reactions are split into forward and backward components such that the reaction set comprises only irreversible reactions. The chance-constrained cell optimization problem has the following constraints:

(6) $$\mathrm{maximize}\left(v_{\mathrm{target}}-\alpha \sum_{j=1}^{M}\left(y_{j}^{u}+y_{j}^{d}\right)\right)$$

s.t.

(7) $$\sum_{j=1}^{M}S_{\mathrm{ij}}v_{j}=0,\forall i\in N$$

(8) $$v_{\mathrm{biomass}}\geq \left(\;,0.01\right)v_{\mathrm{biomass}}^{\max}$$

(9) $$\begin{array}{c} v_{j}\leq \mathrm{SS}U_{j}+y_{j}^{u}\left(F_{j,u}^{-1}\left(\varepsilon \right)-\mathrm{SS}U_{j}\right)\\ +y_{j}^{d}\left(F_{j,d}^{-1}\left(\varepsilon \right)-\mathrm{SS}U_{j}\right),\forall j\in M \end{array}$$

(10) $$v_{j}\geq \mathrm{SS}L_{j}\left(1-y_{j}^{d}\right),\forall j\in M$$

(11) $$\sum_{j=1}^{M}\left(y_{j}^{u}+y_{j}^{d}\right)\leq L$$

(12) $$y_{j}^{u}+y_{j}^{d}\leq 1,\forall j\in M$$

(13) $$\begin{array}{c} y_{j}^{d}+y_{k}^{d}\leq 1,y_{j}^{u}+y_{k}^{u}\leq 1,\\ \forall j\in M,k=j's\;\text{backward}\;\text{counterpart} \end{array}$$

(14) $$y_{j}^{u}\in \left\{0,1\right\},y_{j}^{d}\in \left\{0,1\right\},\forall j\in M$$

The main objective of the problem is to maximize the target reaction flux *v*_*target*_. It is expected that the optimal value of *v*_*target*_ will increase monotonically with *L*, the number of allowed interventions (enzyme up/down-regulation operations). On the other hand, the engineering cost is also expected to increase with the number of interventions. Therefore, the objective function in (6) also includes the term $-\alpha \sum_{j=1}^{M}\left(y_{j}^{u}+y_{j}^{d}\right)$, which imposes a small penalty α for each added intervention, and balances the optimal flux of the target reaction against the number of required interventions. Constraint (7) represents the steady state assumption that the rate of production of each intracellular metabolite is equal to its rate of consumption. Constraint (8) guarantees a minimal growth rate equaling at least 1% of the theoretical maximum of the wild-type (unmodified) organism. A minimal growth rate constraint is required to guarantee that the cell remains viable. This parameter can be adjusted by the user based on the metabolic model, available data and expectations for cell viability, which does not alter the optimization algorithm. To maximize the growth rate while simultaneously maximizing a certain target metabolite, a bi-level optimization with two objectives (maximizing biomass and a target flux) can be applied in place of the constraints (6) and (8). However, linear bi-level programs are NP-hard [18] and there are no efficient algorithms to solve large-scale problems [19]. Constraint (9) sets the upper bound flux capacity for each reaction *j*. Constraint (10) sets the lower bound flux for each reaction *j* to *SSL*_*j*_ (an observed reference state lower bound, if the observation data is available) or zero, based on the value of the binary variable $y_{j}^{d}$. Constraint (11) sets an upper bound on the number of allowed interventions. Inequality (12) ensures that enzyme manipulations are exclusive, i.e. a reaction can be either up- or down-regulated in a solution, but not both. Similarly, constraint (13) guarantees that the forward and backward directions of a reversible reaction are not both up- and down-regulated at the same time. Constraint (14) specifies that the decision variables $y_{j}^{u}$ and $y_{j}^{d}$ can only be 0 or 1.

### Deterministic optimization (Detopt)

The deterministic formulation (DetOpt) can be derived from the CCP formulation by setting *ε* = 0 in (9), i.e. *v*_*j*_ is strictly less than all possible values the random variables $\mathrm{Ca}p_{j}^{u}$ or $\mathrm{Ca}p_{j}^{d}$ can take.

### Monte Carlo-based optimization (MCOpt)

Chance-constrained optimization can be emulated by repeatedly solving the fixed constraint (deterministic) optimization problem in which the constraint parameters ($\mathrm{Ca}p_{j}^{u}$ or $\mathrm{Ca}p_{j}^{d}$) are set to randomly drawn values using a MC sampling procedure for each instance of the problem. The MC sampling requires *a priori* knowledge of the distributions for the flux capacities ($\mathrm{Ca}p_{j}^{u}/\mathrm{Ca}p_{j}^{d}$). The procedure for computing the distributions is described below. Using the randomly drawn set of flux capacities, the capacity constraints become fixed constraints. Effectively, we replace the inequality in (9) with the constraint below:

(15) $$\begin{array}{c} v_{j}\leq \mathrm{SS}U_{j}+y_{j}^{u}\left(X_{j}^{u}-\mathrm{SS}U_{j}\right)\\ \;+y_{j}^{d}\left(X_{j}^{d}-\mathrm{SS}U_{j}\right),\forall j\in M \end{array}$$

where $X_{j}^{u}$ and $X_{j}^{d}$ are the randomly drawn set of flux capacities. Each MC sample, i.e. set of randomly drawn flux capacities, defines an instance of an optimization problem. The solution to this optimization problem is a set of interventions and a corresponding optimal flux value for the target reaction. Repeating the process (sampling and optimization) many times, we obtain a distribution of optimal target flux values.

### Computing capacity distributions

Traditionally, a gene up/down-regulation operation has been modeled as a deterministic event leading to a fold-change in the level of the corresponding enzyme, and hence a fold-change in the flux capacity of the reaction catalyzed by the enzyme. Here, we model enzyme level modification as an uncertain event using a probability distribution. We assume a normal distribution [20] with an average fold-change of *μ* = 6 following gene up-regulation and a spread of *δ =* 6*σ* = 8, where *σ* denotes the standard deviation. The average fold-change value reflects experimental data reported in gene over-expression studies involving mammalian cells, specifically adipocytes [21]. We note that the average fold-change value is a user-specified parameter that can be adjusted to reflect different cell types and experimental data, and thus does not lead to loss of generality. The spread *δ* is chosen so that *μ - δ*/2 > 1, which ensures that the flux capacity after up-regulating the enzyme level is higher than the unmodified state. A decrease in enzyme level, and hence reaction flux capacity, is modeled by a normal distribution *N*_*d*_(*μ*, *σ*^2^) with an average fold-change of *μ* = 0.5 and a spread of *δ* = 1.

Based on the probabilistic interpretation of fold-changes in enzyme levels resulting from gene modifications, we also estimate the resulting reaction flux capacities as probability distributions. We use two different estimation methods depending on whether the model is kinetic or stoichiometric. In the case of a kinetic model, a fold-change in enzyme level is assumed to directly correlate with a fold-change in the maximal reaction velocity (*v*_*j*,max_). Here, the maximal reaction velocity has the same units as reaction flux. Therefore, flux capacity distributions were calculated by simply multiplying the enzyme fold-change distributions with *v*_*j*,max_. In the case of a stoichiometric model, the distributions of flux capacities are approximated using enzyme control flux (ECF) analysis [22]. Briefly, ECF analysis calculates the effect of enzyme level changes on flux distribution based on elementary mode analysis [23] and a power law model for the relationship between reaction flux and enzyme activity. Typically, the ECF problem is underdetermined, and the solution is obtained as a range of minimal and maximal flux for each reaction. We use the maximal flux value as the corresponding reaction flux capacity. The maximal flux values, calculated using sample points from the distributions of enzyme level modifications (*N*_*u*_(*μ*, *σ*^2^) and *N*_*d*_(*μ*, *σ*^2^)), form a capacity distribution.

### Monte Carlo-based evaluation (MCEval) framework

We evaluate CCOpt, DetOpt, and MCOpt using Monte Carlo (MCEval) simulations designed to mimic the expected variations in outcomes when the intervention sets identified by the three different optimization methods are experimentally implemented. For CCOpt and DetOpt, each solution is a single optimal flux of the target reaction and a corresponding set of interventions. The MCOpt solution comprises a distribution of maximal fluxes and their corresponding sets of interventions. To compare these solutions, we perform separate MCEval simulations using the interventions obtained from CCOpt, DetOpt, and MCOpt, and apply flux balance analysis (FBA) [24] with the objective function of maximizing the target flux.

(16) $$\mathrm{maximize}\;v_{\mathrm{target}}$$

s.t.

(17) $$\sum_{j=1}^{M}S_{\mathrm{ij}}v_{j}=0,\forall i\in N$$

(18) $$v_{\mathrm{biomass}}\geq \left(\;,0.01\right)v_{\mathrm{biomass}}^{\max}$$

(19) $$\forall j\in M,v_{j}\leq \left\{\begin{array}{c} \mathrm{SS}U_{j},\mathrm{if}\;\mathrm{reaction}\;j\;\mathrm{is}\;\mathrm{unmodified}\;\\ \;X_{j}^{u},\mathrm{if}\;\mathrm{reaction}\;j\;\mathrm{is}\;\mathrm{up-regulated}\;\\ \;X_{j}^{d},\mathrm{if}\;\mathrm{reaction}\;j\;\mathrm{is}\;\mathrm{down-regulated} \end{array}\right)$$

(20) $$\forall j\in M,v_{j}\geq \left\{\begin{array}{c} \mathrm{SS}L_{j},\mathrm{if}\;\mathrm{reaction}\;j\;\mathrm{is}\;\mathrm{up-regulated}\;\mathrm{or}\;\mathrm{unmodified}\;\\ \;0,\mathrm{if}\;\mathrm{reaction}\;j\;\mathrm{is}\;\mathrm{down-regulated}\; \end{array}\right)$$

In the FBA problem, the flux capacity constraints are drawn from the capacity distributions ($X_{j}^{u}$ and $X_{j}^{d}$ in equation (19)) if the corresponding reaction (enzyme) belongs to the optimized set of interventions. Otherwise, the capacity constraints are set to maximal steady state value (*SSU*_*j*_) calculated for the unmodified reference state. Similar to MCOpt, MCEval repeatedly solves a series of optimization problems to generate a distribution of optimal target flux values. Unlike MCOpt, MCEval does not seek to identify an intervention set reflecting decisions on enzyme activity modification. Rather, each instance of MCEval simply solves for the optimal flux and the corresponding flux distribution based on capacity constraints specified by the CCOpt, DetOpt, or MCOpt solution that is to be evaluated.

## Results and discussion

To assess the benefits and limitations of the optimization methods, we compare their performance using test cases involving both a kinetic and a stoichiometric model. The kinetic model describes the metabolism of Chinese hamster ovary (CHO) cells in fed-batch culture [25]. The stoichiometric model describes the metabolism of adipocytes undergoing differentiation and growth [26].

### CHO cell model

The CHO cell model comprises 24 metabolites and 47 irreversible reactions. The kinetic parameters of the model were previously estimated by fitting the model equations to experimentally obtained metabolite time course data [25]. These parameters are used to estimate the effects of enzyme activity increases and decreases on the corresponding reaction flux capacity distributions. The flux capacity distributions for the adipocyte model are estimated from steady state metabolic flux data obtained in previous studies [27]. Additional details of the model including reaction definitions are provided as Additional file 1. The test objective is the synthesis of a recombinant protein product, a therapeutic antibody.

We first estimate the steady state flux values of a nominal reference state and the corresponding capacity distributions. The reference state fluxes (*SSU*, *SSL*) are estimated through a linear programming formulation that maximizes/minimizes each reaction flux subject to

(21) $$\begin{array}{cc} \;SV & =0;0\leq v_{j}\leq v_{j,\max};v_{j}\\ & =v_{j}^{\text{meas}},j\in \text{MeasuredData} \end{array}$$

where *v*_*j*,max_ is the maximal velocity of reaction *j* and *MeasuredData* is a set of measured exchange flux values for glucose, glutamine, glycine, glutamate and ammonia. The maximal velocities (*v*_*j*,max_) are reported in [25] for only 16 of the 47 reactions in the model that explicitly defined with rate expressions. To calculate the *v*_*j*,max_ values for the remaining reactions, we solve a series of flux maximization problems subject to the 16 pre-defined maximum velocities. The capacities reflecting up/down-regulations of enzyme activities $\mathrm{Ca}p_{j}^{u}/\mathrm{Ca}p_{j}^{d}$ are obtained by multiplying the maximum velocities with the assumed enzyme activity distributions:

(22) $$\mathrm{Ca}p_{j}^{u/d}=v_{j,\max}N_{u/d}\left(\mu ,\sigma^{2}\right)$$

**Figure 3:**
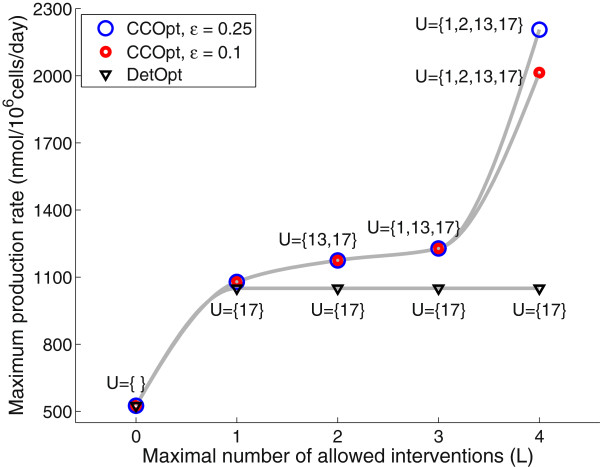
**Maximum antibody production rate and intervention sets obtained by CCOpt and DetOpt using the CHO cell model.** The reactions selected for modification for each intervention set are shown above each data point. The maximum production rates obtained by CCOpt with, *ε* = 0.25, CCOpt with *ε* = 0.1, and DetOpt are shown as blue and red circles and black triangles, respectively. Set U refers to the reactions that need to be up-regulated.

We compare the intervention sets obtained from CCOpt with *ε* = 0.1 and *ε* = 0.25 (representing two choices of conservative and relaxed confidence levels respectively) and those from DetOpt, and evaluate the intervention sets using Monte Carlo simulations (MCEval). In Figure 3, the intervention sets (U for the up-regulation set) identified by each optimization method are shown above their corresponding optimal target flux values. Empty sets represent no identified interventions. For *L* = 1, DetOpt and CCOpt at *ε* = 0.1 and *ε* = 0.25 all select reaction 17, which is the lumped antibody synthesis reaction. For *L* = 2, CCOpt adds reaction 13 to form an intervention set of {13, 17}. Up-regulating reaction 13 increases the synthesis of cysteine, which could be a limiting reactant. As reported in [28], one of the rate-limiting steps of antibody production in CHO cells is the folding and assembly of polypeptides in the endoplasmic reticulum, which requires cysteine residues. For *L* = 3, CCOpt further adds reaction 1, which lumps together several steps in glycolysis. Up-regulating the flux through glycolysis increase the supply of pyruvate for oxidation in the tricarboxylic acid (TCA) cycle, which in turn could provide additional energy for antibody synthesis [29]. For *L* = 4, CCOpt adds reaction 2, which acts to balance the cytosolic redox by oxidizing NADH and possibly relieves feedback inhibition of glycolysis.

Compared to CCOpt, DetOpt predicts smaller maximal antibody synthesis rates (~1000 *nmol/10*^*6*^*cells/day*) due to the conservative choice of reaction flux capacities. The maximal synthesis rate predicted using CCOpt is more than twice the flux predicted by DetOpt (~2200 *nmol/10*^*6*^*cells/day*). The intervention set identified by DetOpt consists of only a single reaction even when the maximal number allowed interventions is raised, indicating that the deterministic method does not fully utilize the degree of freedom available in the problem.

**Figure 4:**
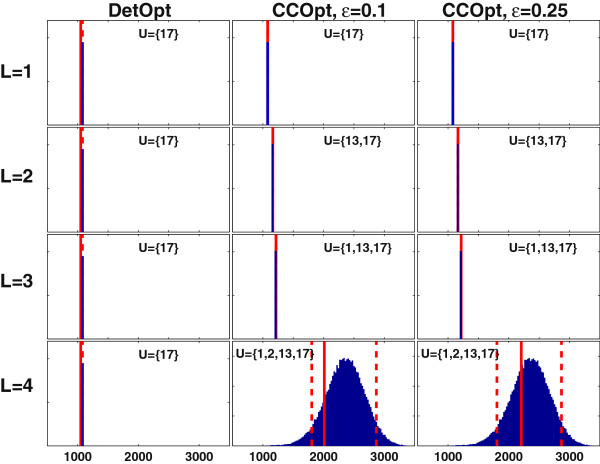
**Monte Carlo sampling based flux balance analysis (FBA) simulations of the intervention sets identified by CCOpt and DetOpt for antibody production using the CHO cell model.** Each panel shows a Monte Carlo distribution of FBA optimized target flux values, with the rows and columns corresponding to different caps on the number of interventions (*L*) and different optimization methods/settings, respectively. The x-axis represents the maximum antibody production rate in units of *nmol/10*^*6*^*cells/day*. The y-axis represents the sampled frequency of an FBA solution. The dashed lines denote the 5^th^ and 95^th^ percentile values, defined as the values below and above which 5 % of the data fall, respectively. A single dashed line indicates that these two percentile values are the same. The solid lines indicate the maximum production rates obtained using CCOpt or DetOpt.

Figure 4 shows the distribution of maximum antibody production rates obtained using MCEval for the intervention sets reported in Figure 3. In all cases, the maximum flux predicted by DetOpt falls outside the probable (5^th^ to 95^th^ percentile) range calculated by MCEval, whereas the maximal flux predicted by CCOpt falls within this range. When only one intervention is allowed (*L* = 1), the selected reaction is the same for CCOpt and DetOpt. However, the flux predicted by CCOpt is higher, and is also more reliable in a probabilistic sense. When the degree of freedom is higher (*L* = 2, 3 and 4), and different intervention sets are selected, MCEval calculates higher probable ranges for the intervention sets identified by CCOpt compared to DetOpt. For example, for *L* = 4, the probable range for CCOpt lies between 1805 and 2870 *nmol/10*^*6*^*cells/day* whereas both the 5^th^ and 95^th^ percentile values for DetOpt are at 1079.

**Figure 5:**
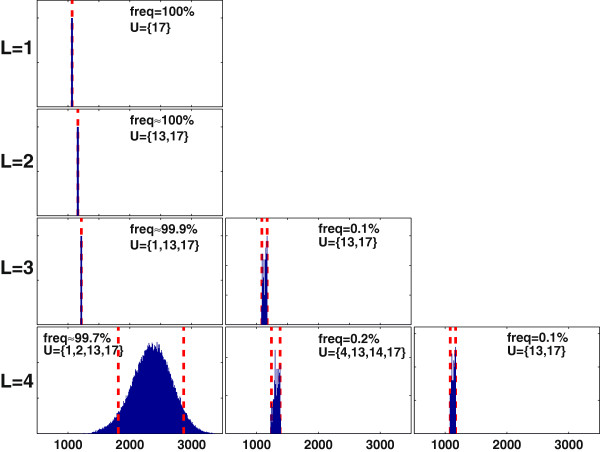
**Monte Carlo sampling based optimization (MCOpt) of antibody production using the CHO cell model.** Each panel shows a MCOpt calculated distribution of target flux values, with the rows and columns corresponding to different caps on the number of interventions (*L*) and different intervention sets, respectively. For *L* = 1 or 2, MCOpt identified only one intervention set. The x-axis represents the maximum antibody production rate in units of *nmol/10*^*6*^*cells/day*. The dashed lines denote the 5^th^ and 95^th^ percentile values. The selection frequency of an intervention set as a fraction of the total pool of MCOpt solutions for a given *L* is shown as a percentile value at the top of each panel.

Figure 5 shows the distribution of solutions resulting from 10^6^ iterations of the Monte Carlo optimization method (MCOpt). MCOpt generates the same solution as CCOpt and DetOpt for *L* = 1 and CCOpt for *L* = 2. For *L* = 3, MCOpt identifies four sets of interventions: {1, 13, 17}, {5, 13, 17}, {13, 17}, and {17}. The first set is dominant at a frequency of 99.86%, and matches the CCOpt solution. For *L* = 4, the trend is the same as *L* = 3, with one dominant solution (frequency > 99%) that matches the corresponding CCOpt solution. This set also corresponds to the highest predicted target flux among all intervention sets comprising four reactions.

In the case of *L* = 4, the aggregate effect of uncertainties in flux capacities is to result in a normally distributed target flux. However, this is not the case for *L* < 4, where the dominant target flux values generated by MCOpt distribute narrowly with nearly zero spread. Moreover, the mean target flux values rise only incrementally from *L* = 1 to 3, suggesting that the probabilistic outcomes accumulate at the lower bound of the probable range due to one or more bottlenecks in the network that are not relieved until all 4 reaction flux capacity modifications are introduced.

**Figure 6:**
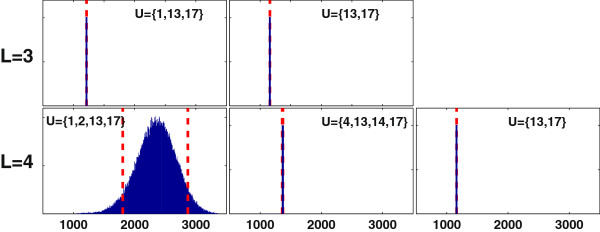
**Monte Carlo sampling based flux balance analysis (FBA) simulations identified by MCOpt for antibody production using the CHO cell model.** Each panel shows a Monte Carlo distribution of FBA optimized target flux values, with the rows and columns corresponding to different caps on the number of interventions (*L*) and different intervention sets, respectively. Results are shown only for *L* = 3 and 4. The x-axis represents the maximum antibody production rate in units of *nmol/10*^*6*^*cells/day*. The y-axis represents the sampled frequency of an FBA solution. The dashed lines denote the 5^th^ and 95^th^ percentile values. A single dashed line indicates that these two percentile values are the same.

Similar to the CCOpt and DetOpt solutions, the MCOpt solutions are evaluated using MCEval (Figure 6). The MCEval results for *L* = 1 and 2 are identical to the MCOpt results for *L* = 1 and 2 shown in Figure 5, respectively. For *L* = 3, MCOpt generates two sets of interventions, where one dominant set is identified with 99.9% frequency. Results of MCEval confirm that this solution ({1, 13, 17}) indeed has a higher probable target flux value. A similar trend is observed for *L* = 4. The set with the highest probable target flux values is identical to the CCOpt solution and the dominant (most frequently identified) MCOpt solution. The probable ranges (5^th^ and 95^th^ percentile values) calculated by MCEval for the MCOpt intervention sets {1, 2, 13, 17}, {4, 13, 14, 17} and {13, 17} are (1805, 2870), (1375, 1389) and (1175, 1175) *nmol/10*^*6*^*cells/day*, respectively. The MCEval simulations produce a normal distribution of target fluxes only for the solution {1, 2, 13, 17}, presumably because only this set of interventions sufficiently relieves the flux capacity bottlenecks in the network. The results of these evaluations indicate that CCOpt and MCOpt essentially identify the same best intervention sets, where CCOpt arrives at the results *without* requiring the sampling run-time cost of MCOpt.

### Adipocyte model

**Figure 7:**
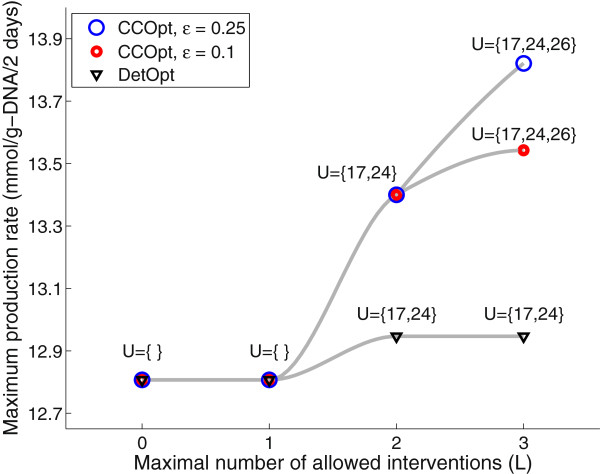
**Maximum tripalmitoylglycerol production rate and intervention sets obtained by CCOpt and DetOpt using the adipocyte model.** The reactions selected for modification for each intervention set are shown above each data point. The maximum production rates obtained by CCOpt with *ε* = 0.25, CCOpt with *ε* = 0.1, and DetOpt are shown as blue and red circles and black triangles, respectively.

In the second case study using the adipocyte model [26], we maximize the production of tripalmitoylglycerol as a representative triacylglycerol (TAG) in adipocyte lipid droplets [30]. This model includes 66 irreversible reactions and 38 metabolites. The details of the model are provided as Additional file 1. Unlike the CHO cell case study, we did not use *v*_*j*,max_ values to estimate the flux capacities and reference state fluxes. Instead, the reference state flux values are calculated by maximizing each reaction subject to a set of measured untreated control data reported in [27]. To estimate the flux capacity distributions, enzyme control flux (ECF) analysis [22] is used, where the analysis calculates the impact of a change in an enzyme’s activity on the steady state flux distribution of the metabolic network. The first step in calculating the distributions is to generate all elementary modes (EMs). For the base adipocyte model, 16,818 EMs were identified using efmtool [31]. In the second step, EM coefficients (EMCs) are calculated through an iterative process. The third step is to estimate the EMCs for a change in enzyme activity. An increase or decrease in enzyme activity is modeled by a normal distribution *N*_*u*_(*μ*, *σ*^2^) or *N*_*d*_(*μ*, *σ*^2^) as described in Methods (Computing capacity distributions). The fourth step is to calculate the flux distributions using the adjusted EMC vectors. Since the enzyme activity change is described by a distribution, multiple flux distributions are calculated. For each reaction in the network, the reaction flux capacity is the set to the maximal flux value of the reaction from the flux distributions. Repeating the third and fourth steps for all reactions generates a statistical distribution of flux capacities for the network. The maximum TG production rate and intervention sets obtained from CCOpt and DetOpt are shown in Figure 7. For both CCOpt and DetOpt, the maximal predicted target flux increases with the number of allowed interventions. As was the case for the CHO cell model, CCOpt predicts a larger maximal flux and generates a more diverse set of solutions compared to DetOpt. In general, DetOpt underutilizes the degrees of freedom available at larger *L* values. For example, the DetOpt solution comprises only 2 interventions when up to 3 interventions are allowed, whereas the CCOpt solution utilizes all 3 allowed interventions. A second general trend is that the smaller sets of interventions are subsets of the larger sets. An interesting observation is that a single intervention (*L* = 1) yields no change in the predicted maximal flux. This is expected, as reactions 17 and 24 are in series, and both are required for TG synthesis. A change in one without a change in the other merely shifts the limiting capacity to the unchanged reaction.

Reaction 17 is a part of the TCA cycle. Reactions 24 and 26 are palmitate biosynthesis and tripalmitoylglycerol biosynthesis, respectively. All three reactions directly impact synthesis of TG, which is formed from esterification of palmitate with glycerol phosphate, with the latter derived from glycerone phosphate. Previous reports [32], including our own work [27], have shown that the addition of long-chain fatty acids stimulates cellular TG accumulation. At first glance, the intervention targets selected by CCOpt appear trivially intuitive. However, other, equally intuitive alternatives also exist, which were not selected. For example, another intuitive intervention to increase net TG accumulation is to down-regulate lipolysis (reaction 27). This intervention was not selected, because the reference (unmodified) state lower bound for reaction 27 is already zero, and a further reduction would have no impact on TG production. In this regard, the optimization results depend not only on the model, but also on the observed reference state.

**Figure 8:**
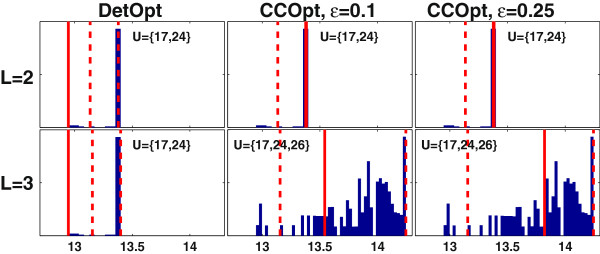
**Monte Carlo sampling based flux balance analysis (FBA) simulations of the intervention sets identified by CCOpt and DetOpt for tripalmitoylglycerol production using the adipocyte model.** Each panel shows a Monte Carlo distribution of FBA optimized target flux values, with the rows and columns corresponding to different caps on the number of interventions (*L*) and different optimization methods/settings, respectively. Results are shown only for *L* = 2 and 3, as setting *L* = 1 failed to produce any solutions (empty sets in Figure 7). The x-axis represents the maximum production rate in units of *mmol/g-DNA/2 days*. The y-axis represents the sampled frequency of an FBA solution. The dashed lines denote the 5^th^ and 95^th^ percentile values. The solid lines indicate the maximum production rates obtained using CCOpt or DetOpt.

As was the case for the CHO cell model, the results of CCOpt more closely match the results of MCEval simulations compared to DetOpt (Figure 8). Since neither DetOpt nor CCOpt identified any solutions for *L* = 1, MCEval simulations are not shown. For *L* = 2 and 3, the maximal fluxes predicted by DetOpt (13 *mmol/g-DNA/2 days*, shown as solid lines) lie at the lower end of the distributions generated by MCEval. In contrast, the maximal fluxes predicted by CCOpt consistently fall in the probable (5^th^-95^th^ percentile) range (shown as dashed lines) of the MCEval distributions. For *L* = 3, the 95^th^ percentile value obtained from MCEval simulations of the CCOpt intervention set is significantly larger than the 95^th^ percentile value obtained from MCEval simulations of the DetOpt intervention set. Additionally, the flux values predicted by CCOpt with and are both in the probable range as calculated by MCEval.

**Figure 9:**
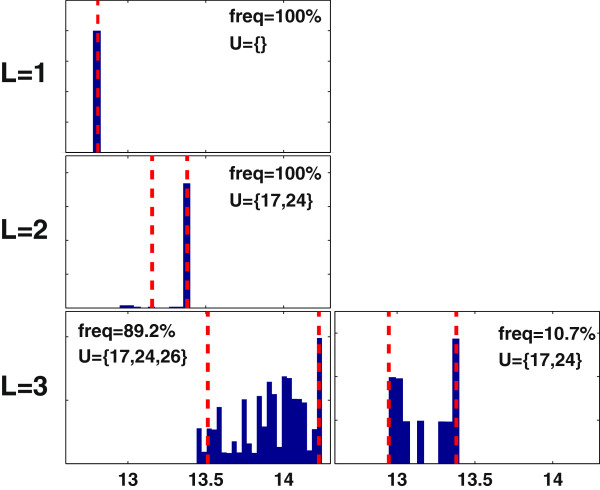
**Monte Carlo sampling based optimization (MCOpt) of tripalmitoylglycerol (TG) synthesis using the adipocyte model.** Each panel shows a MCOpt calculated distribution of target flux values, with the rows and columns corresponding to different caps on the number of interventions (*L*) and different intervention sets, respectively. For *L* = 1 or 2, MCOpt identified only one intervention set. The x-axis represents the maximum TG synthesis rate. The dashed lines denote the 5^th^ and 95^th^ percentile values. The selection frequency of an intervention set as a fraction of the total pool of MCOpt solutions for a given *L* is shown as a percentile value at the top of each panel.

**Figure 10:**
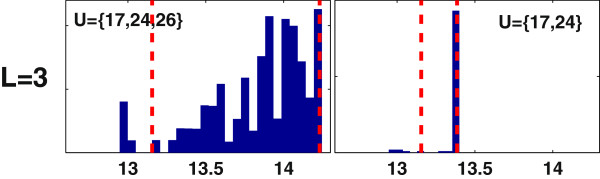
**Monte Carlo sampling based flux balance analysis (FBA) simulations of the intervention sets identified by MCOpt for tripalmitoylglycerol production using the adipocyte model.** Each panel shows a Monte Carlo distribution of FBA optimized target flux values, with the rows and columns corresponding to different caps on the number of interventions (*L*) and different intervention sets, respectively. The x-axis represents the maximum production rate in units of *mmol/g-DNA/2 days*. The y-axis represents the sampled frequency of an FBA solution.

Applying MCOpt to the adipocyte model generates one solution for *L* = 1 and 2 and two solutions for *L* = 3 (Figure 9). The solutions with the highest frequency are identical to the CCOpt solutions. These solutions are {}, {17, 24} and {17, 24, 26} for *L* = 1, 2, and 3, respectively, and occur with 100%, 100% and 89.2% frequency. Of the two MCOpt solutions for *L* = 3, the dominant solution has the higher probable target flux values, which is consistent with the results of MCEval simulations (Figure 10).

### Computational complexity and scalability of methods

Our optimization problems (CCOpt, MCOpt and DetOpt) are formulated as mixed integer linear programming (MILP). A MILP problem requires a subset of variables to take on integer values, while the other variables can take on non-integer values. This problem is NP-hard [33], and thus it is unlikely that there exists an efficient (polynomial-time in the size of the model) algorithm to obtain a globally optimal solution. In the present study, we implemented our optimization methods (CCOpt, MCOpt and DetOpt) using the GNU Linear Programming Kit (GLPK) [34] in MATLAB. The runtime of our computational experiments solving the MILP problems was on the order of a few seconds on a Core i5 2.53 GHz CPU.

In addition to the scalability issue inherent to MILP problems, another computational challenge lies in estimating the flux capacity distributions. For the stoichiometric model of this study, we used enzyme control flux analysis (ECF) [22] to obtain these distributions. The ECF method in turn relies on elementary mode (EM) analysis, which can be applied to metabolic models comprising < ~100 reactions, but remains intractable for genome-scale models. An alternative strategy is to model the fold-change in flux capacity, i.e. enzyme activity, resulting from a gene expression modification using a probability distribution, e.g. a normal distribution. This strategy requires knowledge of maximal enzyme velocities (v_max_). If these parameters are not known, they may be estimated from FBA, which has been demonstrated on genome-scale models.

These types of limitations, while not trivial, are comparable to other computational strain design methods. For example, bi-level optimization, used in OptKnock [4], is also NP-hard [35], and thus can be intractable for large-scale problems. As an NP-hard problem, the runtime grows exponentially with the number of allowed reaction modifications [5]. Methods that rely on EM analysis [14-16,36] face a similar limitation as our capacity estimation problem, as the analysis is generally only practical for small to mid-scale models. Methods based on local search [5] or metaheuristics [13,37] are computationally less prohibitive than MILP, and likely offer the best alternative for large-scale problems. On the other hand, these methods cannot guarantee global solution optimality, and may arrive at solutions that are far from exact.

## Conclusions

This study investigates three distinct ways of capturing uncertainty about parameter values when formulating an optimization problem with the objective of identifying targets for enzyme activity adjustments that maximize the production of a desired molecule. The three approaches are chance-constrained programming (CCOpt), Monte Carlo sampling-based solution of the uncertain problem (MCOpt), and deterministic optimization based on worst-case assumptions (DetOpt). Evaluation of the approaches for two test cases (CHO cell and adipocyte models) using Monte Carlo simulations (MCEval) shows that a more sophisticated probabilistic approach such as CCOpt has several advantages compared to a conservative conventional approach like DetOpt. Chance-constrained programming explores a larger portion of the solution space and is able to find a more diverse set of options. Additionally, CCOpt consistently outperforms DetOpt in terms of predicting the more likely maximum of the objective function value. Comparisons of the intervention sets from CCOpt and DetOpt using MCEval shows that the maximal fluxes predicted by CCOpt was always in the probable (5^th^-95^th^ percentile) range calculated by MCEval, whereas the maximal fluxes predicted by DetOpt typically lies outside of this range. When compared to the sampling-based optimization approach (MCOpt), CCOpt consistently finds the solution most frequently selected by MCOpt, but at a fraction of the computational cost (seconds vs. days).

The CCOpt formulation can be readily extended to capture other types of uncertainties, such as biological variability in measured data and cell transfection efficiency, making CCOpt an effective technique for probabilistic strain optimization.

## Abbreviations

CCOpt: Chance-Constrained optimization; DetOpt: Deterministic optimization; MCOpt: Monte Carlo-based optimization; MCEval: Monte Carlo Evaluations; CCP: Chance-constrained programming; CDF: Cumulative distribution functions; ECF: Enzyme control flux; EM: Elementary modes; FBA: Flux balance analysis; CHO: Chinese hamster ovary; TCA: Tricarboxylic acid; TG: Tripalmitoylglycerol; GLPK: GNU Linear Programming Kit; MILP: Mixed integer linear programming.

## Competing interests

The authors declare that they have no competing interests.

## Authors’ contributions

SH and KL conceived the idea of applying probabilistic methods to gene modifications. MO provided probabilistic expertise. All authors participated in formulating the problem. MY implemented the methods, generated all the figures and drafted the manuscript. All authors read and approved the final manuscript.

## Supplementary Material

Additional file 1 Detailed models of CHO cell and adipocyte. Click here for file
